# *Globicatella sanguinis*—A Literature Review of Case Reports

**DOI:** 10.3390/medicina61112048

**Published:** 2025-11-17

**Authors:** Carmen Valerica Rîpă, Roxana Gabriela Cobzaru, Luminita Smaranda Iancu, Miruna Raluca Rîpă, Gabriela Popescu, Alexandra Maștaleru, Andra Oancea, Maria Magdalena Leon

**Affiliations:** 1 Grigore T. Popa University of Medicine and Pharmacy Iași, 700115 Iași, Romania; ripa.carmen@umfiasi.ro (C.V.R.); luminita.iancu@umfiasi.ro (L.S.I.); mg-rom-32810@students.umfiasi.ro (M.R.R.); gabriela.popescu@umfiasi.ro (G.P.); alexandra.mastaleru@umfiasi.ro (A.M.); andra.oancea@umfiasi.ro (A.O.); maria.leon@umfiasi.ro (M.M.L.); 2 Department of Preventive Medicine and Interdisciplinarity (IX)—Microbiology, Grigore T. Popa University of Medicine and Pharmacy Iași, 700115 Iași, Romania; 3 Department of Medical Specialties I, Grigore T. Popa University of Medicine and Pharmacy Iași, 700115 Iași, Romania; 4 Clinical Rehabilitation Hospital, 700661 Iași, Romania

**Keywords:** *Globicatella sanguinis*, infection, rare, case reports, antimicrobial therapy, narrative review

## Abstract

*Globicatella sanguinis* is a rare and distinct Gram-positive coccus, first described in 1992, and part of the normal human microbiota. Although infrequent, it can cause serious infections such as urinary tract infections, meningitis, and endocarditis, particularly in vulnerable populations. Due to its atypical biochemical profile and frequent misidentification by conventional diagnostic systems, accurate identification is essential to avoid inappropriate treatment. This narrative review analyzes 21 published case reports involving 51 patients, with many cases linked to invasive procedures or immunosuppression. The review emphasizes the need for molecular diagnostic tools and individualized antimicrobial therapy, while also identifying gaps in current knowledge that warrant further investigation.

## 1. Introduction

Bacteria belonging to the genus *Globicatella* are Gram-positive cocci, facultative anaerobes, catalase-negative, and non-alpha hemolytic [1]. *Globicatella sanguinis (G. sanguinis)* is a rare and distinct genus that is part of the normal flora in humans, first described by Collins et al. in 1992 [2]. Therefore, it is part of the normal flora in humans, but it can cause infections in the urinary, meningeal, and cardiac areas [1,2]. *Globicatella* may be isolated from blood cultures, urine, cerebrospinal fluid or other clinical specimens. Growth in 6.5% NaCl broth combined with a negative leucine aminopeptidase (LAP) test is a useful distinguishing characteristic. Phenotypic identification using commercial systems such as Vitek2, API and Phoenix is frequently inaccurate, often resulting in misidentification as streptococci or enterococci. Consequently, conventional laboratory methods may be insufficient for definitive identification. The most reliable approaches are 16S rRNA gene sequencing and matrix-assisted laser desorption/ionization time-of-flight mass spectrometry (MALDI-TOF MS), provided the reference database includes the genus *Globicatella* [3]. Recognition of *G. sanguinis* as a potential human pathogen is important, since its atypical antimicrobial susceptibility pattern and frequent misidentification may lead to inappropriate therapy, while documenting and reporting existing cases remains essential to raise awareness, expand reference databases, and clarify its true epidemiological and clinical significance.

## 2. Materials and Methods

This review aims to synthesize the available case reports on *Globicatella sanguinis*, focusing on diagnostic challenges, antimicrobial susceptibility trends and clinical outcomes.

Given the descriptive purpose of the study, the authors focused on case reports, which provide clinical, paraclinical, and therapeutic details about individual cases of *Globicatella sanguinis* infection. The literature search was conducted on three platforms (PubMed, Web of Science, Embase) using the keyword combination “*Globicatella sanguinis*” and “case report(s)”, without restrictions regarding year or geographical region. Additionally, the inclusion criteria targeted English-language articles available in open access that described cases of human infection. Articles published in languages other than English, book chapters, books, systematic review or meta-analysis articles, letters to the editor, animal studies, and articles for which the full text was not available were excluded.

After carefully evaluating the titles, we continued with reading the abstracts, eliminating the articles that did not seem suitable for the proposed topic, so that in the end, the reviewers would complete the full reading of the articles (Table 1).

Being a narrative review based on case reports, no statistical analyses were performed by the authors. The data presented (age, sex, outcomes) are descriptive and reflect the information reported in the original articles.

## 3. Discussions

### 3.1. Prevalence and Prevention

Despite the increased recognition made possible by advanced diagnostic technology, *G. sanguinis* remains rare, with only sporadic reports and small case series to date. Twenty-one articles published between 2001 and 2025 were discovered following the literature study. Among these, six originate from the USA, five from India, two from Japan, and one each from the UK, Korea, Morocco, Saudi Arabia, Denmark, Germany, Nigeria, and France. In total, 51 patients were included, ranging in age from a few hours to 94 years old. Of these, 30 were female, 15 were male, and sex information was not available for 6 patients. Out of the 51 patients, the cause of infection could not be determined in 36 cases. Among the known causes of *G. sanguinis* infection, surgical interventions or invasive procedures, trauma, cat bites, an immunocompromised status, or complications related to pregnancy and childbirth can be mentioned.

The general data of the articles included in the review can be seen in Table 2.

Infections caused by *Globicatella sanguinis* are rare clinical events, but their importance is increasing in vulnerable populations. The cases reviewed indicate that infections often occur in patients with major predisposing factors such as immunosuppression, extreme age (newborns or elderly) [1,5,8,10,12,15,18,19], or following surgical interventions and trauma. At present, no evidence supports human-to-human transmission of *G. sanguinis*. Therefore, preventive measure is extrapolated from general infection control principles, including strict aseptic technique during invasive procedures, careful monitoring of immunocompromised patients, and neonatal hygiene in complicated births. These recommendations should be regarded as general rather than pathogen-specific. Several of the analyzed cases illustrate the direct link between the onset of infection and a medically invasive context: hip replacement [3], cardiac implant [20], ventriculoperitoneal shunt [6,22], or knee arthroplasty [13]. In these situations, primary prevention consists of strict adherence to sterility protocols during invasive procedures. In immunocompromised patients [3,5], preventive measures include careful monitoring for early clinical signs of infection and limiting exposure to potential sources of bacterial contamination.

Another important preventive aspect stems from recognizing situations that favor secondary infections—open wounds—as is the case with the patient bitten by a cat [17]. In these cases, secondary prevention consists of early diagnosis, biological sample collection, and prompt adjustment of empirical antimicrobial therapy based on culture results. Furthermore, in newborns and infants, prevention could focus on careful monitoring of complicated births—prematurity, premature rupture of membranes, presence of meconium in amniotic fluid [1,18]—and implementing neonatal hygiene measures. Therefore, there are no specific preventive measures for infection with this pathogen described in the literature, but general infection prevention strategies are the key solution. Regarding the patients included in the study, it cannot be stated that there is a pattern, but cases where the infection was caused by trauma were represented only in male patients [9,14,17].

Regarding the distribution of the infection among age groups and gender, numerous findings can be drawn from the analysis of the cases included in the study. The sex of only 45 out of the 51 cases was identified, with 30 being female and 15 being male. Data on the age at the time of infection was available for only 26 women among these patients. Consequently, by categorizing them by age, 6 patients aged 0–14 years, 1 woman aged 15–25 years, 5 women aged 25–64 years, and 14 women aged over 65 years were identified. The average age of the women was 53.92 years. Excluding pediatric cases, the average age of the patients in the study is 69.6 years (20 patients). In the case of men, their age is known for only 14 of them. Regarding age groups, four male patients aged 0–14 years, no male patients aged 15–25 years, six patients aged 25–64 years, and four aged over 65 years can be mentioned. The average age of the men is 46.64 years; excluding minors, the average age increases to 64.1 years (10 men). The available case reports suggest a possible predominance among older female patients; however, this observation should be interpreted with caution given the small number of cases and heterogeneity of the data. Regarding the evolution of cases, Table S1 in the supplementary data shows that three patients had an unfavorable outcome, followed by death, this evolution being marked by the condition of the newborn or severe associated comorbidities, even though the treatment initiated was the one obtained following the antibiogram analysis.

Therefore, when discussing prevention, it is important first to consider the extreme age groups, as they are much more susceptible to infections caused by rare bacteria; additionally, it appears that women are significantly more prone to these types of infections. However, future studies are needed to confirm or refute this aspect.

### 3.2. Diagnosis

Regarding diagnostic methods, in 28 patients confirmation was achieved through DNA sequencing. In 12 patients, the pathogen was identified by blood cultures, in 6 cases by cerebrospinal fluid cultures, and in 1 case by urine culture. In a single patient, synovial tissue culture was required, while in two additional cases the diagnosis was established by PCR and by inoculation on culture medium, respectively, without specification of the biological specimen used. As can be seen from the cases included in the review, the diagnosis can be established through several methods; however, the main difficulty lies in differentiating *G. sanguinis* from *Aerococcus, Enterococcus, and Streptococcus viridans.* In contrast to streptococci and enterococci, *G. sanguinis* does not grow at temperatures of 10 degrees Celsius; however, it can develop in a medium containing 6.5% NaCl and does not produce leucine aminopeptidase (LAP) [12,18,23]. Another specific characteristic of *Globicatella* is their microscopic appearance: *G. sanguinis* forms chains, unlike aerococci which tend to group in clusters and tetrads. Given that standard laboratory methods cannot definitively differentiate between infection with *G. sanguinis* and *Globicatella sulfidifaciens*, the use of amplification techniques such as PCR and 16S rRNA gene sequencing is recommended for precise phenotypic characterization. MALDI-TOF MS was employed for microbial identification, whereas antimicrobial susceptibility testing was carried out using the Vitek 2 system [5,12,18,20] (Table 3).

### 3.3. Treatment

The treatment used for this infection in the patients whose cases were included in this review was quite heterogeneous, and the following agents can be mentioned: fluoroquinolones (moxifloxacin, levofloxacin); penicillin (penicillin, amoxicillin, piperacillin); macrolides (roxithromycin); third-generation cephalosporins (cefoperazone); beta-lactamase inhibitors (sulbactam, tazobactam); aminoglycosides (amikacin, gentamicin); glycopeptides (vancomycin, teicoplanin); linezolid; and meropenem.

Given that it is difficult to identify *Globicatella sanguinis* microbiologically, it is important to compare its antibiotic susceptibility with that of other species that appear similar, such as *Aerococcus, Enterococcus,* and the *viridans group Streptococcus*. Research indicates that *G. sanguinis* is predominantly susceptible to penicillin, amoxicillin, chloramphenicol, and levofloxacin, yet might exhibit inconsistent resistance to cefotaxime and metronidazole. *Aerococcus urinae*, on the other hand, is usually sensitive to β-lactams and vancomycin but resistant to trimethoprim–sulfamethoxazole. *Enterococcus faecium* exhibits inherent resistance to β-lactams and cephalosporins, while vancomycin-resistant enterococci complicate treatment alternatives. *Streptococcus viridans* typically maintains susceptibility to penicillin, although macrolide resistance is being reported with increasing frequency. Consequently, species misidentification may result in unsuitable empirical treatment, potentially yielding detrimental clinical consequences [4,24].

Regarding treatment, there is no exact data on medication that can be routinely used for *Globicatella* infection. However, some studies show that there is an increased sensitivity to penicillin, amoxicillin, chloramphenicol, and levofloxacin [3,12]. Despite this, among the cases included in the review, penicillin, amoxicillin, and levofloxacin therapy is mentioned in only 7 cases, while out of the 51 cases mentioned, the treatment followed by the patients is not mentioned in 31 of them. Regardless of the literature data, it is imperative to determine the pathogen’s antibiotic susceptibility prior to initiating targeted therapy. For this microorganism, the most commonly used methods to establish minimum inhibitory concentration are the Vitek 2 advanced system or disc diffusion methods [9]. In infections caused by opportunistic pathogens, establishing appropriate therapy is essential to reduce the risks associated with the infection [12,18].

## 4. Strengths and Limitations

Because *G. sanguinis* infection is quite rare, there is a relatively small number of published cases on this topic but also a considerable heterogeneity in the clinical data. The lack of diagnostic and therapeutic standards limits the comparative interpretation of the results. However, the review of these cases brings this rare pathogen and its impact on patient health back to the attention of the medical community while also opening up new perspectives for future research.

## 5. Conclusions

*Globicatella sanguinis* remains a rare but clinically relevant pathogen, particularly in vulnerable populations such as neonates, elderly patients, and immunocompromised individuals. Through the analysis of 21 case reports published between 2001 and 2025, encompassing 51 patients from diverse geographical regions, this review highlights several important findings that contribute to the current understanding of this microorganism. Firstly, the demographic distribution suggests a potential predominance of infections in female patients, especially those over the age of 65. Although the sample size is limited, it underscores the importance of recognizing predisposing factors such as surgical intervention, trauma, and immunosuppression, which were frequently associated with the onset of infection. These findings reinforce the need for heightened clinical vigilance in such contexts. From a diagnostic perspective, the review confirms that conventional laboratory methods often fail to accurately identify *G. sanguinis*, leading to frequent misclassification as *Aerococcus, Enterococcus,* or *Streptococcus viridans.* This misidentification has direct therapeutic implication, such as antimicrobial susceptibility; advanced molecular techniques such as 16S rRNA sequencing and MALDI-TOF MS are therefore essential for reliable identification and appropriate treatment.

Therapeutically, the cases reviewed reveal a heterogeneous approach, with no standardized regimen. However, sensitivity to penicillin, amoxicillin, and levofloxacin was noted in several reports, suggesting these agents may be effective when guided by susceptibility testing. The frequent omission of treatment details in published cases highlights the need for more comprehensive reporting to inform clinical practice.

In conclusion, this review not only consolidates existing knowledge about *Globicatella sanguinis* but also identifies patterns and gaps that warrant further investigation. The findings emphasize the importance of accurate diagnosis, awareness of risk factors and individualized antimicrobial therapy. Future research should aim to establish standardized diagnostic and therapeutic protocols and explore the pathogen’s epidemiology in greater depth.

## Figures and Tables

**Table 1:** The process of identifying the articles included in the review.

Platform	Query	Filters	Results	Paper Included	Paper Excluded and Reasons for Exclusion
Web of science	“globicatella sanguinis”	1992–2025	33	21	12—other theme from than this review
	“globicatella sanguinis” AND “case report”	1992–2025	9	7	1—erratum 1—letter to editor
Embase	‘globicatella sanguinis’/exp OR ‘globicatella sanguinis’	1992–2025	39	21	17—other theme from than this review 1—study on animals
	(‘globicatella sanguinis’/exp OR ‘globicatella sanguinis’) AND ‘case report’	1992–2025	21	20	1—study on animals
Pub Med	(“globicatella sanguinis” [Supplementary Concept] OR “globicatella sanguinis” [All Fields]) AND (1992:2025[pdat])	1992–2025	24	16	4—other theme from than this review 1—other language than English 1—no full text version available 2—study on animals
	((“globicatella sanguinis” [Supplementary Concept] OR “globicatella sanguinis” [All Fields]) AND (“case reports” [Publication Type] OR “case report” [All Fields])) AND (1992:2025 [pdat])	1992–2025	12	7	1—other language than English 1—no full text version available 2—study on animals 1—other theme from than this review

**Table 2:** General data of the articles included in the review.

Author and Year	Country	Sex and Age	Cause of Infection	Onset Symptoms/Clinical Examination	Initial Treatment	Final Treatment	Diagnostic Test
P. L. Shewmaker et al.; 2001 [4]	USA	Age—between 1 and 85 years Sex: 17 cases—female (±60 years) 6 cases—male (±53.25 years) 5 cases—not available	Not mentioned	14 cases—not available 3 cases—bacteremia-specific symptoms 3 cases—urinary tract infection symptoms 6 cases—sepsis-specific symptoms 1 case—endocarditis-specific symptoms 1 case—meningitis-specific symptoms	Not mentioned	Not mentioned	All cases—DNA strain 22 cases—blood 2 cases—cerebrospinal fluid (CSF) 4 cases—urine
RJ Abdul-Redha et al.; 2007 [5]	Denmark	Case 1—female, 23 years Case 2—female, 82 years Case 3—male, 56 years	Case 1—high susceptibility for infectious disease Case 2—No data Case 3—no data	Case 1—high temperature, mild dyspnea, thoracic pain Case 2—dehydration, urosepticaemia Case 3—mild dyspnea, erysipelas, fever	Case 1—Cefuroxime Case 2—no data Case 3—Cefuroxime	Case 1—Penicillin Case 2—Penicillin Case 3—Roxithromycin and Moxifloxacin	Blood cultures
I. Seegmuller et al.; 2006 [6]	Germany	Female, 69 years	Implantation of ventriculoperitoneal shunt.	Somnolent, vomiting and clinical sign of meningitis.	Ceftriaxone	Ceftriaxone	Culture from cerebrospinal fluid (CSF)
G. Hery-Arnaud et al.; 2010 [7]	France	Female, 56 years	Not mentioned	Vomiting, headache, gait disorders, memory impairment	Cefotaxime and Fosfomycin	Amoxicillin	Culture from cerebrospinal fluid (CSF)
M. Matsunami et al.; 2011 [8]	Japan	Male, 94 years	Not mentioned	Back pain, fever.	Ampicillin and Sulbactam, then Vancomycin	Penicillin G.	Blood cultures Urine cultures
N.Jain; 2012 [9]	India	Male, 70 years	Fall from height	High-grade fever Local infection Unconsciousness	Amoxicillin Clavulanic acid	Levofloxacin Cefoperazone Sulbactam Amikacin (i.v.)	Culture from cerebrospinal fluid (CSF)
A. O. Miller et al.; 2016 [3]	USA	Case 1—female, 72 years Case 2—female, 54 years	Case 1—hip arthroplasty Case 2—immunosuppression	Case 1—pain and dysfunction of the hip Case 2—abdominal pain, fatigue, fever	Case 1—vancomycin Case 2—vancomycin	Case 1—vancomycin Case 2—linezolid	Case 1—cultures from synovial tissue Case 2—blood culture
U. Devi et al.; 2016 [10]	India	Female, 2 days old	Not mentioned	Seizure Refusal to feed	Cloxacillin Amikacin	Not mentioned	Culture from cerebrospinal fluid (CSF)
S. Snagli et al.; 2018 [11]	USA	Female, 64 years	Not mentioned	Seizures Encephalopathy Hypotension Hypothermic	Broad spectrum antibiotics for septic shock	Broad spectrum antibiotics for septic shock	Blood cultures
S. Takahashi et al.; 2018 [12]	Japan	Female, 87 years	UTI	High fever Hematuria	Ceftriaxone	Meropenem Ampicillin	Blood cultures
K. Ahn et al.; 2018 [13]	Korea	Female, 76 years	Knee replacement surgery	Knee pain, inflammatory syndrome	Vancomycin and Levofloxacin	Vancomycin and Levofloxacin	Blood cultures
B. Gupta et al.; 2021 [14]	India	Male, 9 years	Left eye trauma	Pain, redness, discharge, vision diminution	Vancomycin Tobramycin (topical) Ciprofloxacin (i.v.)	Teicoplanin (i.v.)	Microbiological examination (culture)
H. Skali et al.; 2022 [15]	Morocco	Female, 5 months old	Not mentioned	Fever, vomiting, seizures, and feeding problems	Biantibiotherapy based on 3^rd^-generation cephalosporins and Gentamicin	Not mentioned	Cerebrospinal fluid culture
E.M. Elkholy, 2023 [16]	Saudi Arabia	Female, 38 years	C-section 27 days before presentation	Dyspnea, orthopnea, headache, left knee joint arthralgia	Vancomycin Ceftriaxone	Penicillin G Gentamycin	Blood culture
N.K. Jones; 2023 [17]	UK	Male, 48 years	Cat bite	Cellulitis	Doxycycline, Ciprofloxacin, Metronidazole (p.o.) Vancomycin (i.v.)	Vancomycin, Ciprofloxacin, Metronidazole (i.v.)	PCR
H.K. Obaro et al., 2023 [18]	Nigeria	Female, 3 h old	Premature delivery due to spontaneous rupture of the membrane	Difficulty in breathing, inability to feed	Gentamicin, Cefuroxime	Not mentioned	Blood cultures
N. Santuka et al., 2023 [19]	India	Male, newborn (day 1)	Hospital-acquired infection	Convulsion Crying disorders Hypoglycemia	Ampicillin Cefotaxime Amikacin Linezolid	Linezolid	Blood cultures
I. Afon et al.; 2023 [20]	USA	Male, 63 years	Implantation of a cardiac loop	Right-sided weakness, slurred speech, headache	Not mentioned	Not mentioned	Blood cultures
B. Huynh et al.; 2024 [21]	USA	Male, 62 years	Immunosuppression	Edema and weakness	Vancomycin, Cefepime, Flagyl	Vancomycin, Piperacillin/Tazobactam	Blood cultures
S. Varma et al.; 2024 [22]	India	Male, 36 years	Implantation of ventriculoperitoneal shunt.	Vomiting Fever Pus discharge from the shunt tract	Amikacin Cefoperazone-sulbactam	Linezolid	Cerebrospinal fluid cultures
M.L. Hyatt; 2025 [1]	USA	Neonate	Thick meconium in the amniotic fluid due to prolonged rupture of membranes	Breathing issue, poor tone	Ampicillin, Gentamicin	Increased dose of Ampicillin Gentamicin	Blood cultures

**Table 3:** Diagnostic algorithm for *Globicatella sanguinis* infections.

Step	Diagnostic Stage	Details
1	Patient history	Trauma, surgery, invasive procedures, immunosuppression.
2	Clinical examination	Local signs of infection.
3	Imaging	MRI, CT, ultrasound—for deep-seated infections.
4	Microscopy	Gram stain: Gram-positive cocci in chains.
5	Culture	Blood agar 5%, chocolate agar—slow growth, alpha-hemolysis weak or absent
6	Biochemical differentiation	LAP negative, growth in 6.5% NaCl, growth at 10 °C—helps distinguish from viridans streptococci.
7	Automated phenotypic systems	VITEK 2, API 20 Strep
8	Proteomic identification	Matrix-assisted laser desorption ionization time-of-flight mass spectrometry—MALDI-TOF MS—accuracy depends on library coverage.
9	Molecular confirmation	16S rRNA PCR, sodA gene sequencing—gold standard for rare or ambiguous isolates
10	Antimicrobial susceptibility	Vitek 2, E-test—variable susceptibility.

MRI—magnetic resonance imaging; CT—computed tomography; LAP—leucine aminopeptidase; API 20 Strep—Analytical profile index 20 Strep; PCR—polymerase chain reaction; 16S rRNA—16S ribosomal RNA; E-test—epsilometer test.

## Supplementary Materials

The following supporting information can be downloaded at https://www.mdpi.com/article/10.3390/medicina61112048/s1: Table S1: Supplementary data of cases included in the review.
