# Supplementary material for: Globicatella sanguinis—A Literature Review of Case Reports
Source: Medicina (Kaunas). 2025 Nov 17;61(11):2048. doi: 10.3390/medicina61112048 (PMC12654734; doi:10.3390/medicina61112048)
Supplement: Supplementary file 1 [file medicina-61-02048-s001.zip › medicina-3955525-supplementary.pdf]

Table S1. Supplementary data of cases included in the review

Author and Year	Sex and age	Associated pathologies	Antimicrobial Susceptibility Results	Initial treatment	Final treatment	Diagnostic confirmation	Evolution
P. L. Shewmaker et al.; 2001[4]	Age - between 1-85 years<br>Sex:<br>17 cases – female (±60 years)<br>6 cases – male (±53.25 years)<br>5 cases – not available	Not mentioned	Antibiotic susceptibility tests: All isolates were susceptible to 2 of the 12 antimicrobial agents, amoxicillin and vancomycin.	Not mentioned	Not mentioned	Rapid ID 32 strep	Not mentioned
RJ Abdul-Redha et al.; 2007 [5]	Case 1 – female, 23 years<br>Case 2 – female, 82 years<br>Case 3 – male, 56 years	Case 1 - intravenous drug use, Right-sided endocarditis, hepatitis C<br>Case 2 – Alzheimer's disease, hypertension<br>Case 3 – Crohn's disease, atrial fibrillation	Case 1, 2 – blood culture and susceptibility test – penicillin<br>Case 3 – blood cultures – susceptibility test cefuroxime, roxithromycin and moxifloxacin (allergic to penicillin)	Case 1 – Cefuroxime<br>Case 2 – no data<br>Case 3 – Cefuroxime	Case 1 – Penicillin<br>Case 2 – Penicillin<br>Case 3 – Roxithromycin and Moxifloxacin	Case 1-3 - 16S rRNA Sequencing and API Rapid ID32 Strep	Case 1-3 – favorable
I. Seegmuller et al; 2006 [6]	Female, 69 years	Ventriculoperitoneal Shunt	Antibiotic susceptibility tests: cefotaxime (MIC ≤ 0.5 µg/ml)	Ceftriaxone	Ceftriaxone	Rapid ID 32 strep, Phoenix system PMIC/ID-56<br>16S rRNA sequence analysis	Favorable

G. Hery-Arnaud et al; 2010 [7]	Female, 56 years	Not mentioned	Antibiotic susceptibility tests: amoxicillin (MIC ≤ 0.016 μg/ml)	Cefotaxime and Fosfomycin	Amoxicillin	16S rRNA sequencing	Favorable
M. Matsunami et al; 2011 [8]	Male, 94 years	Hyperlipidemia; hypertension; dementia; osteoporosis	Antibiotic susceptibility tests: Penicillin G (MIC = 0.25 μg/ml)	Ampicillin and Sulbactam, then Vancomycin	Penicillin G.	Vitek 2<br>16 rRNA sequencing	Favorable
N.Jain; 2012 [9]	Male, 70 years	Craniectomy	Antibiotic susceptibility tests: sensitive to amoxicillin + clavulanic acid, vancomycin, levofloxacin, chloramphenicol, but resistant to penicillin, ampicillin, erythromycin, clindamycin and tetracycline	Amoxicillin Clavulanic acid	Levofloxacin<br>Cefoperazone<br>Sulbactam<br>Amikacin (i.v.)	Vitek 2	Favorable
A. O. Miller et al; 2016 [3]	Case 1 – female, 72 years<br>Case 2 – female, 54 years	Case 1 – Obesity; gastric lap banding; lymphedema.<br>Case 2 – obesity; diabetes mellitus; gastric by-pass; recurrent cystitis	Antibiotic susceptibility Etest: penicillin G (Case 1 - MIC = 0.094 μg/ml; Case 2 - MIC = 0.125 μg/m); Vancomycin (Case 1 - MIC = 0.125 μg/ml; Case 2 - MIC = 0.19 μg/m)	Case 1 – vancomycin<br>Case 2 – vancomycin	Case 1 – vancomycin<br>Case 2 – linezolid	Case 1&2 – Partial 16S rRNA Sequencing, MALDI-TOF-MS	Case 1 – favorable, but after 9 months patient died<br>Case 2 – favorable
U. Devi et al; 2016 [10]	Female, 2 days old	Not mentioned	Antibiotic susceptibility: sensitive to levofloxacin, ofloxacin, vancomycin and linezolid.	Cloxacillin Amikacin	Not mentioned	16S rRNA sequencies	Favorable
S. Snagli et al; 2018 [11]	Female, 64 years	Hypertension; Proctocolitis	Not mentioned	Broad spectrum antibiotics for septic shock	Broad spectrum antibiotics for septic shock	Vitek	Favorable

S. Takahashi et al; 2018 [12]	Female, 87 years	Endocarditis; UTI	Antibiotic susceptibility: Meropenem (MIC = 0.25 mg/ml); Ampicillin (MIC ≤ 0.25 mg/ml);	Ceftriaxone	Meropenem Ampicillin	Rapid ID 32 Strep,16S rRNA sequencing	Favorable
K. Ahn et al; 2018 [13]	Female, 76 years	Hypertension Rheumatoid arthritis	Antibiotic susceptibility tests: vancomycin (MIC = 0.25μg/ml); levofloxacin (MIC = 0.25μg/ml);	Vancomycin and Levofloxacin	Vancomycin and Levofloxacin	16S rRNA sequencing	Favorable
B. Gupta et al; 2021 [14]	Male, 9 years	Corneal abscess; Endophthalmitis	Antibiotic susceptibility test: sensitivity to amoxicillin, ampicillin, vancomycin, erythromycin, cotrimoxazole, chloramphenicol, and Teicoplanin AND no sensitivity ciprofloxacin	Vancomycin Tobramycin (topical) Ciprofloxacin (i.v.)	Teicoplanin (i.v.)	MALDI-TOF -MS and Vitek 2	Not mentioned
H. Skali et al; 2022 [15]	Female, 5 months old	Posterior fossa tumor	Antibiotic susceptibility to levofloxacin, moxifloxacin, vancomycin, teicoplanin, linezolid, erythromycin, clindamycin, gentamycin.	Biantibiotherapy based on 3rd generation cephalosporins and Gentamicin	Biantibiotherapy based on 3rd generation cephalosporins and Gentamycin	MALDITOF-MS	Unfavorable - death
E.M. Elkholy, 2023 [16]	Female, 38 years	C-section 27 days before presentation	Antibiotic susceptibility tests: penicillin MIC < 0.12 μg/ml);	Vancomycin Ceftriaxone	Penicillin G Gentamycin	Blood cultures	Favorable
N.K. Jones; 2023 [17]	Male, 48 years	Obesity	Ampicillin, MIC ≤0.016 mg/L; cefotaxime, MIC 0.004	Doxycycline, Ciprofloxacin, Metronidazole (p.o.) Vancomycin (i.v.)	Vancomycin, Ciprofloxacin, Metronidazole (i.v.)	16S rRNA gene sequence API Rapid ID 32 Strep analysis MALDITOF-MS	Favorable

			mg/L; penicillin, MIC ≤0.016 mg/L; linezolid, MIC 1.0 mg/L; ciprofloxacin, MIC 0.032 mg/L; vancomycin, MIC 0.25 mg/L and moxifloxacin, MIC 0.016 mg/L.				
H. K. Obaro et al, 2023 [18]	Female, 3-hour-old	Preterm neonate	Antibiotic susceptibility tests: sensitive to gentamicin, cefuroxime, ceftriaxone and ceftazidime	Gentamicin, Cefuroxime	Not mentioned	Vitek 2	Unfavorable - death
N. Santuka et al, 2023 [19]	Male, newborn (day1)	Large for gestational age	Antibiotic susceptibility tests: sensitivity to to ampicillin, doxycycline, tetracycline, linezolid, and vancomycin.	Ampicillin Cefotaxime Amikacin Linezolid	Linezolid	BACT/ALERT and VITEK 2, bioMérieux	Favorable
I. Afon et al; 2023 [20]	Male, 63 years	Uncontrolled HTN, HLD, HIV, ESRD and DM type 2	Not mentioned	Not mentioned	Not mentioned	Blood cultures	Not mentioned
B. Huynh et al; 2024 [21]	Male, 62 years	Cachexia, CVA, dysphagia, multiple unstageable decubitus ulcers	Not mentioned	Vancomycin, Cefepime, Levofloxacin	Vancomycin, Piperacillin/Tazobactam	Maldi Biotyper Sepsi typer	Unfavorable – comfort care
S. Varma et al; 2024 [22]	Male, 36 years	Ventriculoperitoneal shunt	Vitek 2: Sensitive to Vancomycin and Linezolid	Amikacin Cefoperazone-sulbactam	Linezolid	MALDI-TOF MS	Favorable
M.L. Hyatt; 2025 [1]	Neonate	Suspected Meconium Aspiration Syndrome	Culture sensitivities were never received	Ampicillin, Gentamicin	Increased dose of Ampicillin Gentamicin	Blood cultures	Favorable
